# Commentary: Combination of nucleated red blood cells and inflammatory biomarkers (PCT and CRP) for predicting sepsis and septic shock in children

**DOI:** 10.3389/fcimb.2025.1699391

**Published:** 2025-11-28

**Authors:** Hai-Bo Zheng, Guo-Ming Zhang

**Affiliations:** Department of Laboratory Medicine, Shuyang Hospital, The Affiliated Shuyang Hospital of Xuzhou Medical University, Shuyang, Jiangsu, China

**Keywords:** C-reactive protein, procalcitonin, nucleated red blood cell, sepsis, septic shock

## Introduction

Sepsis and septic shock remain leading causes of morbidity and mortality among critically ill children, underscoring the urgent need for reliable biomarkers to improve early diagnosis and risk stratification (Singer et al., 2016). Conventional markers such as procalcitonin (PCT) and C-reactive protein (CRP) have shown moderate diagnostic accuracy but limited predictive capacity (Wacker et al., 2013). Nucleated red blood cells (NRBCs) have recently emerged as potential indicators of systemic stress and hypoxia (Stachon et al., 2007). However, methodological rigor—including appropriate control groups, accurate reporting of sensitivity/specificity with valid confidence intervals, and transparent modeling—is critical to ensure the clinical applicability of novel biomarker panels (Rutjes et al., 2005; Collins et al., 2015).

## General comments

Li et al. conducted a retrospective study of 121 children (80 with sepsis and 41 with septic shock) and concluded that the use of nucleated red blood cells (NRBCs), when combined with procalcitonin (PCT) and C-reactive protein (CRP), yielded superior diagnostic accuracy, with an AUC of 0.956 (Pu et al., 2025). While the authors address an important clinical problem, several methodological and reporting concerns limit the robustness of their findings.

First, the study objective and title mismatch are considered. The study cohort comprises patients already meeting sepsis criteria; ROC analyses compare *septic shock* versus *sepsis* (severity stratification), rather than *sepsis* versus *non-sepsis*. Consequently, reported AUCs quantify the discrimination of severity within an already diseased cohort, not the ability to *predict* or *diagnose sepsis* among children at risk. The manuscript’s title and several statements imply predictive diagnostic performance for sepsis more broadly; this inference is unsupported without inclusion of uninfected controls or a prospective prediction design. The authors should either (a) revise the title and conclusions to reflect severity stratification among septic patients or (b) provide analyses including appropriate non-infected or unselected controls to support claims of sepsis prediction (Rutjes et al., 2005; Singer et al., 2016).

Second, there are internal inconsistencies in the ROC results. We noted discrepancies in the results of the ROC analyses. The AUC for PCT was 0.868 (95% CI 0.818–0.912), whereas Table 3 lists 0.886. Similar inconsistencies appear for the CRP and combined models across text, tables, and figures (e.g., Figure 4. The ROC curve of the CRP level shown in Figure 4 was compared with the sensitivity of 78.1% shown in Table 3. The specificity of the CRP in the figure was less than 50%, which is far from the specificity value of the CRP in Table 3 of 76.7%.) (Pu et al., 2025), and a similar situation was reported in Li’s previous paper (Figure 5) (Li et al., 2023). Even small mismatches undermine confidence. We recommend rechecking all ROC outputs, applying consistent rounding rules, and providing exact values in the supplementary materials. Reporting sensitivity, specificity, PPV, NPV, and likelihood ratios with 95% CIs would enhance clarity (Pu et al., 2025).

Third, there are internal inconsistencies and units. The PCT AUC is reported as 0.868 in the ROC test but 0.886 in Table 3; this discrepancy should be reconciled. The text also lists a PCT cutoff of “≥ 7.785 mg/L,” whereas the Methods section cites thresholds of PCT at 0.05 ng/mL; unit harmonization is essential. Moreover, NRBCs are “manually counted per high-power field,” yet the cutoff is given simply as “≥ 3” without a measurement basis (cells/HPF? cells per 100 WBC? per µL), precluding reproducibility and external use. Inter/intra-observer reliability and device calibration (or correlation with automated analyzers) were not provided. This finding is also inconsistent with a previous study in which the NRBC was calculated via the Sysmex XN hematology analyzer (Li et al., 2023).

Fourth, comparative analysis of PCT is insufficient. PCT has a competitive AUC and prognostic value, yet subgroup ROC plots and formal pairwise DeLong comparisons between PCT and the proposed combination are incomplete. To substantiate superiority claims, we provide subgroup ROC curves (with 95% CI bands) and DeLong test statistics (DeLong et al., 1988) for PCT versus the combination.

Finally, transparency in the combined model is lacking. The construction of the “CRP+PCT+NRBC” index is not specified (logistic regression, weighted score, or summation). Moreover, no internal validation or calibration was performed. Without adherence to the TRIPOD reporting standards, reproducibility remains limited (Collins et al., 2015).

## Discussion

The inclusion of NRBCs in risk assessment for critically ill children is a promising avenue and may ultimately provide clinicians with additional tools to improve severity stratification. However, the current study has several major limitations, including inconsistencies in diagnostic reporting, the absence of non-infected control groups, incomplete comparative analysis of PCT, and insufficient transparency in the combined biomarker panel. These issues substantially undermine the robustness of the findings and restrict their clinical applicability. To increase the credibility and utility of the results, we strongly recommend that the authors publish corrected diagnostic tables with raw data, explicitly define the study’s target condition, present full ROC and pairwise statistical comparisons, and clearly describe the construction and validation of the combined biomarker model in line with established reporting standards.
